# Microbial pathways for advanced biofuel production

**DOI:** 10.1042/BST20210764

**Published:** 2022-04-11

**Authors:** John Love

**Affiliations:** The BioEconomy Centre, Biosciences, College of Life & Environmental Sciences, The University of Exeter, Stocker Road, Exeter EX4 4QD, U.K.

**Keywords:** biocatalysts, biofuels, metabolic engineering, microbiology, synthetic biology

## Abstract

Decarbonisation of the transport sector is essential to mitigate anthropogenic climate change. Microbial metabolisms are already integral to the production of renewable, sustainable fuels and, building on that foundation, are being re-engineered to generate the advanced biofuels that will maintain mobility of people and goods during the energy transition. This review surveys the range of natural and engineered microbial systems for advanced biofuels production and summarises some of the techno-economic challenges associated with their implementation at industrial scales.

## Introduction

**Figure 1.**
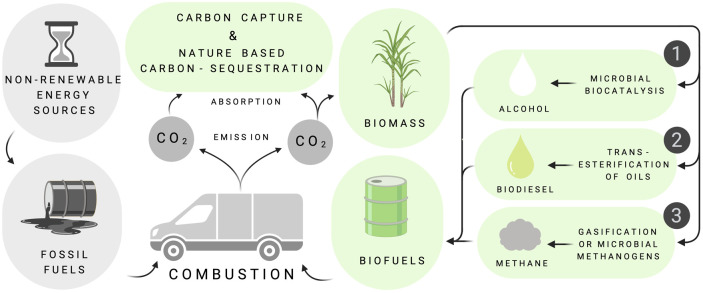
Routes to decarbonisation. Decarbonisation aims to reduce the environmental impact of fossil fuel use, either by the substitution of fossil energy in the form of coal, petroleum and natural gas by fuels derived from biomass or by direct C-capture and nature-based C-sequestration strategies (termed ‘Bioenergy with Carbon Capture Solutions (BioCCS or BECCS) [4] which are currently used to offset emissions in the form of carbon credits. In the transport sector, the market penetration of emission-free electric vehicles is accelerating but is heavily reliant on C-free electrical generation capacity and accessible rapid charging infrastructures which may be difficult to implement in non-urban or more isolated settings, or in developing countries, hence the ongoing (and currently increasing) need for biofuels. Current transport biofuels include alcohols (1), lipid-derived biodiesels (2) and biomass-derived or microbially generated combustible gasses such as [bio]methane and H_2_ (3) that can be catalytically converted to sustainable synthetic fuels. Figure drawn using Biorender software.

Since the industrial revolution, the use of fossilised biomass for energy and chemicals has altered the natural carbon (C) cycle, with major impacts now felt worldwide [1]. Liquid transport fuels are a convenient form of concentrated energy with established production standards, storage facilities and distribution networks. Transport consumes ∼65% of global petroleum production and accounts for approximately one third of total energy consumption, annually [2]. Biomass-derived transport fuels (Figure 1) are therefore key to the decarbonisation of the transport sector [3].

**Figure 2.**
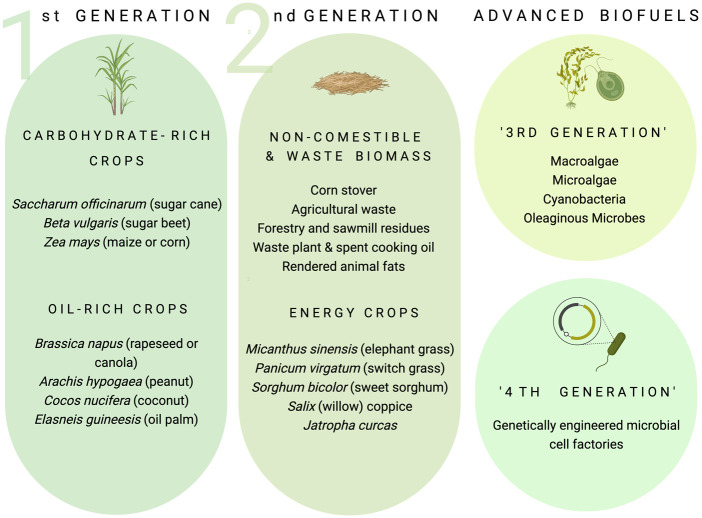
Classification of transport biofuels. First (1G) and second generation (2G) biofuels describe the origin of the biomass used to manufacture the fuel. 1G biofuels are produced from carbohydrate- or oil-rich food crops whereas 2G biofuels exploit a wider variety of non-comestible and lignocellulosic energy crops, waste lignocellulosic biomass (agricultural, forestry or sawmill residues) inedible or waste plant oils, spent cooking oil or rendered animal fats. Advanced biofuels, sometimes referred to as third- or fourth- generation (3G or 4G) biofuels, encompass a range of alternative biomass sources or combustible molecules derived from microbes, notably microalgae or oleaginous yeasts, or from metabolically engineered microbial cell factories. Figure drawn using Biorender software.

Biofuels encompass solid, liquid or gaseous combustible materials that are derived from, and produced by, living organisms [5] (Figure 2). Current, commercial biofuels include microbially produced alcohols, principally ethanol (C_2_H_5_OH) from *Saccharomyces cerevisiae* fermentation and *n*-butanol (C_4_H_9_OH) from *Clostridium* bacteria or chemical conversion of ethanol, that are blended with gasoline. Biodiesels are produced by the catalytic conversion of tri-acyl-glycerides (TAG) with alkaline catalysts (e.g. NaOH, KOH or CH_3_NaO) and methanol, yielding fatty acid mono-alkyl esters (FAMEs or crude biodiesels) and glycerol. Plant oils can also be chemically hydrotreated to generate hydrogenated vegetable oils (HVOs or ‘renewable diesel fuels’ [6]) that are de-oxygenated, linear alkane hydrocarbons which can directly substitute for fossil-derived automotive gas oil [7].

‘Advanced biofuels’ encompass a range of alternative combustible molecules or biomass that are typically derived from microbes. The term ‘third generation’ (3G) is often used by researchers in academia to describe both the biomass and the fuel precursors derived from microalgae and heterotrophic microbes other than those commonly used in commercial 1G and 2G biofuel production [8,9]. Biofuels from genetically engineered microalgae, bacteria and fungi have, by extension, been dubbed ‘fourth generation’ (4G) [10–12].

Following a summary of the microbial metabolisms used for the production of current, commercialised biofuels, this review presents an overview of the variety of microbial routes to innovative biofuels and some, though by no means all, of the techno-economic challenges associated with generating advanced microbial biofuels at industrial scales.

## Renewable natural gas and synthetic fuels

Renewable Natural Gas (RNG) can be produced by direct, thermochemical gasification of biomass feedstocks to generate syngas, a mix of CO, CO_2_ and H_2_, followed by chemical methanation [13], or by anaerobic fermentation (also termed ‘anaerobic digestion’; AD) of organic matter such as manure, food waste or sewerage sludge. AD generates a mix of 50–75% CH_4_ and 25–50% CO_2_, with traces of H_2_, siloxanes and SH_2_. Chemical upgrading is then performed to remove the contaminants and raise the methane content to above 90%, resulting in commercial ‘biomethane’ or ‘biogas’ [14].

Microbial methanogenesis involves 4 phases, each of which is performed by different groups of mesophilic (20–45°C) or thermophillic (50–70°C) microbes that are all present in the reactor consortium [15]. In phase 1, termed the hydrolytic phase, facultatively anaerobic bacteria degrade organic polymers from the feedstock, releasing soluble substrates. These substrates are then metabolised during the second, acidogenic phase to produce a mix of compounds including short-chain volatile fatty acids, organic acids, alcohols, ketones, and different gasses (CO_2_, NH_3,_ SH_2_ and H_2_). In the third phase, acetogenesis, the products of acidogenesis are metabolised by H_2_-producing, acetogenic and homoacetogenic bacteria. The acetogenic bacteria, represented by *Syntrophomonas*, *Syntrophospora*, *Syntrophobacter*, *Fusobacterium* and *Paleobacter*, metabolise ≥ C_3_ organic acids, ethanol and aromatic compounds into acetate (CH_3_COO^−^), formate (CH_2_O_2_), CO_2_ and H_2_. The homoacetogenic bacteria metabolise the substrates produced in the acidogenic phase or can use H_2_ and CO_2_ via the acetyl-CoA pathway to produce acetate (CH_3_COO^−^). Finally, methanogenesis is undertaken by three groups of methanogenic archaebacteria; the hydrogenotrophs, the aceticlasts and the methylotrophs [16]. During hygenotrophic methanogenesis CO_2_ is reduced to CH_4_ and H_2_O, using 4 H_2_. In aceticlastic methanogenesis, acetate is cleaved in the presence of H^+^ to form CH_4_ and CO_2_ and estimates suggest that this accounts for ∼70% of the CH_4_ generated. In methylotropic methanogenesis, methylated C_1_ compounds (e.g. methanol, methylamines and di-methyl sulphide) are converted to CH_4_, CO_2_ and H_2_O.

Like methanated syngas produced by thermochemical biomass gasification, RNG may be used directly for energy or as precursors for catalytic re-forming to produce advanced fuel hydrocarbons [13,17,18]. Additionally, AD may be combined with other microbial processes to generate additional biofuels. For example, the fibrous material resulting from AD of cow manure can be pre-treated as a lignocellulosic feedstock and subsequently fermented to ethanol [19].

## Alcohol biofuels

### Ethanol

Worldwide, approximately 100 billion litres of ethanol (C_2_H_5_OH) are produced annually through the anaerobic fermentation of hexoses from 1G biomass by the yeast, *S. cerevisiae*. Although *S. cerevisiae* can also metabolise sugar aerobically via respiration, ‘Crabtree-positive’ yeast strains primarily use the fermentative pathway even when O_2_ is present in the medium [20].

The respiratory and fermentative (ethanogenic) pathways start with glycolysis which requires one molecule of glucose and two molecules of NAD^+^ to yield two molecules of ATP and two molecules of pyruvate. During respiration, the pyruvate enters the tricarboxylic acid cycle and is completely metabolised to CO_2_ and ATP. Under anaerobic conditions, the pyruvate is metabolised to equimolar quantities of acetaldehyde and thence to ethanol and CO_2_ and the recycling of NAD^+^. The industrial production of ethanol uses highly adapted yeast strains that can withstand the stresses imposed by large-scale bioreactor cultivation [21,22], including temperature [23], pH [24], the presence of metabolic inhibitors from treated plant biomass [25] and the accumulation of ethanol in the medium [26], such that sugar to ethanol conversion can be as high as 90% and ethanol concentrations of 20% readily achieved [27].

The production of 2G ethanol from lignocellulose first requires saccharification of the biomass to its constituent hexose and pentose monomers by various pre-treatments including chemical hydrolysis using concentrated acid, alkali, ionic liquids, or eutectic solvents; thermochemical hydrolysis that employs mechanical extrusion (milling); pyrolysis; microwaving; steam- or CO_2_-based explosion; and biological hydrolysis using lignin-degrading fungi (notably *Trichoderma reesei*), eubacteria and archaea, or purified enzyme mixes [28–35]. Lignocellulose pre-treatment yields a slurry containing high concentrations of both hexose and pentose sugars, the latter which cannot be metabolised by *S. cerevisiae* [36]. Pentoses, however, can be metabolised by other yeasts, including *Pichia spp.*, *Candida spp.*, *Schizosaccharomyces spp.*, *Pachysolen spp.* and *Kluyveromyces spp.* [37], enbling the formulation of co-cultures capable of hexose and pentose fermentation [21,38,39]. Alternatively, hybrid yeast strains of *S. cerevisiae* and species capable of pentose fermentation, produced by protoplast fusion and interspecific genome shuffling, may be used to ferment both pentoses and hexoses, increasing the efficiency of the conversion from biomass to ethanol [40,41].

### *n*-Butanol

*n*-Butanol is a promising alternative to ethanol because of its higher energy density, higher lubricity, lower viscosity, lower corrosiveness and lower hygroscopicity [42,43]. *n-*Butanol is produced by chemical conversion of ethanol using Mg or Al mixed oxides or hydroxyapatite catalysts [44], or by fermentation of 1G and 2G feedstocks via the Acetone-Butanol-Ethanol (ABE) pathway of *Clostridium* bacteria [45]. *Clostridium* are obligate anaerobes and only four species produce sufficient quantities of butanol to be industrially relevant: *C. acetobutylicum* (the model for ABE fermentation), *C. beijerinckii* (a potential candidate for lignocellulosic conversion to butanol), *C. saccaroperbutylacetonicum* and *C. saccharoacetobutylicum* [44]. ABE fermentation occurs in two stages: In stage 1, growing bacteria produce acetic and butyric acids from acetyl-CoA via a suite of enzymatic steps involving acetyl-CoA acetyltransferase (AtoB), 3-hydroxybutyryl-CoA dehydrogenase (Hbd), crotonase (Crt), butyryl-CoA dehydrogenase (Bcd), and alcohol/aldehyde dehydrogenase (AdhE2). In stage 2, the bacteria enter stationary phase during which they accumulate granulose, form endospores and re-assimilate the metabolic acids to form acetone, butanol, ethanol, CO_2_ and H_2_.

**Figure 3.**
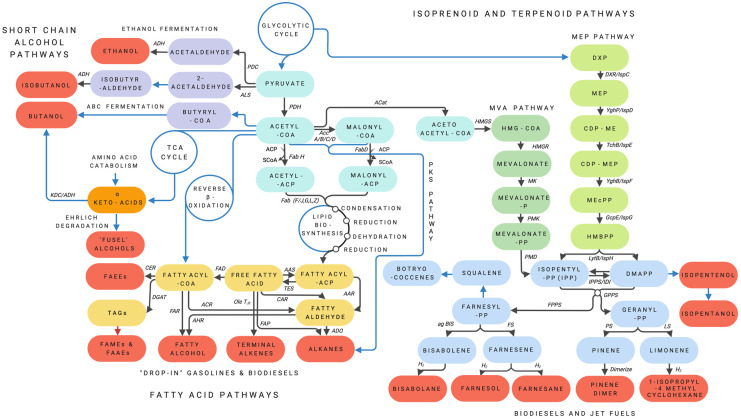
Natural and engineered metabolic pathways to biofuels. Schematic diagram of natural and engineered pathways to biofuels and biofuel precursors in microbial platforms. Note, not all pathways are simultaneously present in any single microbe. Metabolic pathways are coloured thus — Core metabolism: light blue. Short chain alcohol pathways: purple. The Ehrlich degradative pathway to fusel alcohols: orange. Fatty acid metabolism: yellow. Isoprenoid MVA and MEP pathways: green. Terpenoid synthetic pathways: blue. The various biofuels are highlighted in red. Black arrows represent enzymatic transformations, with the enzymes or enzyme classes indicated. Blue arrows represent multiple enzymatic transformations in the metabolic pathways indicated. The red arrow represents abiotic, chemical conversion of TAGs to FAMEs and FAAEs. Abbreviations for enzymes are: PDC, Pyruvate dehydrogenase complex; ALS, Acetolactate (acetohydroxyacid) synthase; ADH, Alcohol dehydrogenase; KDC, α-keto acid decarboxylase; PDH, Pyruvate dehydrogenase; FabH, 3-oxoacyl-[acyl-carrier-protein] synthase; FabD, Malonyl CoA-acyl carrier protein transacylase; Acc A/B/C/D, Acetyl-CoA carboxylase; ACat, Acetyl transferase; AAS, Acyl-ACP synthase; TES, Thioesterase; AAR, Acyl-ACP reductase; FadD, acyl-CoA synthase; OleT_JE_, CYP152L1(cytochrome P450 fatty acid peroxygenase); FAP, fatty acid photodecarboxylase; CAR, carboxylic acid reductase; CER, Wax ester synthase; DGAT, Diglyceride acyltransferase; FAR, Fatty acid reductase; ACR, acyl-CoA reductase; AHR, Aldehyde reductase; ADO, aldehyde deformylating oxygenase; HMGS, 3-hydroxy-3-methylglutaryl-CoA synthase; HMGR, HMG-CoA reductase; MK, mevalonate kinase; PMK, phosphomevalonate kinase; PMD, phosphomevalonate decarboxylase; DXR/ispC, DXP reductoisomerase; IPPS, IPP isomerase; IDI, Isopentenyl-diphosphate delta isomerase; GPPS, geranyl diphosphate synthase; PS, pinene synthase; LS, limonene synthase; agBIS, bisabolene synthase; FS, farnesene synthase.

Several techno-economic challenges associated with the biocatalytic production of butanol at scale remain, including the cost of the fermentative process itself compared with production by chemical catalysis, low butanol yields due to the cytotoxicity of butanol and difficulties in improving or engineering *Clostridium* directly [46–48]. Consequently, the genes encoding the *Clostridium* butanol production pathway have been engineered in the more tractable and facultatively anaerobic *E. coli* [49,50] enabling pathway improvements through enzyme engineering [51]. Finally, as an alternative, ‘advanced’ *n*-butanol can also be generated through engineered decarboxylation and reduction in short-chain α-keto acids (Figure 3) by an α-keto acid decarboxylase/alcohol dehydrogenase combination [52]. However, these improved or alternative metabolic routes can only be commercially realised if associated with a more profound understanding of the molecular mechanisms of *n-*butanol tolerance and the consequent development of new host strains or biocatalysts in which that tolerance is significantly enhanced [53].

### Engineering the production of alternative short chain and higher alcohols

The need to replace petroleum has also focussed on the microbial production of alternative short chain (≤C_4_) alcohols, notably *n*-propanol (C_3_H_7_OH) and isopropanol (CH_3_CHOHCH_3_) [54]. While *n*- and iso-propanol are combustible, their production cost and marginal energy gain relative to ethanol and the advantages of butanol as a fuel mean that n-propanol is probably better suited as a precursor chemical or fuel additive than as an alternative bulk biofuel.

Higher alcohols, characterised by C-chains longer than C_4_ are promising biofuels but, like *n*- or iso-propanol, do not accumulate naturally in microbes and so must be produced by metabolic engineering [55]. Higher alcohols may also be produced from amino-acid catabolism via the Ehrlich pathway (Figure 3) to yield so-called ‘fusel alcohols’. In this pathway, valine, leucine, isoleucine, methionine, and phenylalanine may be transaminated to α-keto acids and converted by alcohol dehydrogenases into fusel alcohols or, depending on the cellular redox state, to carboxylic acids. The C-chain of the fusel alcohols is equivalent to that of the amino-acid substrate minus 1 C [56].

Fatty alcohols, notably 1-octanol, are attractive targets for biofuels as they may be used to increase the physio-chemical properties of fuel blends [57]. Long-chain acyl-CoAs or acyl-ACPs are reduced to fatty alcohols via fatty-aldehyde intermediates by various acyl-CoA/ACP reductases (Figure 3). Alternatively, free fatty acids may be reduced to fatty alcohols by carboxylic acid reductases (CAR [58]) or by the Lux C/D/E complex from bioluminescent bacteria [11], also via fatty-aldehydes. The presence throughout the phylogeny of these different enzyme families provides opportunities for metabolic engineering and production of fatty-alcohols with specific C-chain lengths, in heterologous hosts including *E. coli* and yeasts [59–61]. However, the over-production of fatty-alcohols for biofuels is not trivial; obstacles include metabolic repression that inherently act to limit productive titres, accurate C-chain length modification and the functionalisation of the fatty acid intermediates into the desired product(s) [62] and remains an area of intense research.

## Third generation microbial oils, mycodiesels and algal biofuels

Oleaginous microbes are defined as those that can accumulate over 20% of their biomass as TAG [63], usually in intracellular vesicles. Bacteria store energy principally as poly-β-hydroxy-butyrates or -alkanaoates, not TAGs, so ‘oleaginous microbes’ are primarily represented by some yeast and filamentous fungi (e.g. *Candida, Cryptococcus, Debaryomyces, Lipomyces, Rhodotorula, Rhodosporidium, Saccharomycodes, Trichosporon* and *Yarrowia*), and microalgae.

### Oleaginous yeasts

Oleaginous yeasts (the model for which is *Yarrowia lipolytica*) are amenable to large-scale fermentation and present an attractive platform for generating natural and modified long-chain lipids and TAGs from both 1G and 2G feedstocks [64,65]. TAG accumulation in oleaginous yeasts is dependent on culture conditions such as a low C:N ratio, temperature, pH, [O_2_] and the concentration of trace elements and inorganic salts [64,66]. In response to nitrogen limitation, AMP-desanimase degrades intracellular adenosine monophosphate (AMP) to inosine monophosphate and NH_4_^+^ for use as an alternative source of nitrogen. The rapid decrease in [AMP] suppresses the activity of NAD- and NADP-dependent isocitrate dehydrogenase, altering the Krebs cycle and resulting in the accumulation of iso-citric acid and citrate within mitochondria. Citrate is then exported from the mitochondria in exchange for cytoplasmic malate, and cleaved by ATP citrate lyase, an enzyme exclusively found in oleaginous microorganisms, to form oxaloacetate and acetyl-CoA which is the precursor of lipid biosynthesis by reverse β-oxidation [64,66]. The fatty acids produced in this manner are subsequently stored as TAG (Figure 3) that may be extracted and converted to biodiesel.

Economically efficient production of microbial biodiesels requires economically efficient fermentation, harvesting and processing i.e. the use of cheap raw materials with minimal fermentation inhibitors [67], simple monitoring and adjustment of the culture media C : N ratio, and cost-effective processes for lipid extraction and subsequent processing [68]. For even high-producing oleaginous yeasts, separating the biomass from the culture medium and disruption of the yeast cell wall to extract the storage lipids is expensive. Consequently, microorganisms that secrete rather than store the lipids they produce can simplify downstream processing [12] and increase efficiency. To that end, lipid secretion has been achieved in modified strains of *Trichosporon cutaneum, Candida lipolytica*, and acetyl-CoA synthase deletion mutants of *S. cerevisiae* [69,70], and, although the precise mechanism of secretion remains unknown, continued research has the potential to lower processing costs and increase the potential of oleaginous yeasts as a renewable source of lipid for biodiesel.

### Mycodiesels

Mycodiesels [71] describe a range of volatile organic compounds produced by some endophytic fungi, notably *Gliocladeum roseum* [72] and *Ascoryne sarcoides* [73,74], and include acetic acid esters of straight chained alkanes and higher alcohols that might readily be converted to drop-in biofuels. Currently basic research into mycodiesels appears somewhat quiescent, however the molecular pathways to these promising metabolic products are largely uncharacterised and may yet be exploited as resources for engineering innovative metabolic pathways in more tractable microbial hosts.

### Advanced biodiesels from microalgae

Microalgae are a polyphyletic grouping comprising over 40 000 identified species of photoautotrophic microbes living in a range of environments, from Antarctic ice to the edges of volcanic hot-springs and from fresh to hypersaline waters. The production of advanced biofuels from microalgae has several theoretical advantages compared with terrestrial biomass: Microalgae may be cultured on marginal or non-arable land thus circumventing the food *vs.* fuel controversy of G1 biofuels [75] and in brackish, saline or wastewater thereby avoiding use of increasingly precious freshwater [76,77]. Microalgal cell walls are composed of cellulose, shorter polysaccharides and protein and only a few species possess lignin [78]. Consequently, microalgal cultures yield a more homogenous biomass than that produced by multicellular plants, which limits post-harvest waste and simplifies the fermentation of non-oleaginous species grown as a (3G) biomass crop or the second-stage processing of algal residues following extraction of oil or other, higher value compounds for which the algae may be exploited. Most importantly, depending on the species, location of the algal culture facility and culture conditions, microalgae have substantially higher biomass and lipid productivity compared with terrestrial feedstocks [79–81]. For example, an oleaginous microalga that produces 30% of its fresh biomass as oil can achieve biodiesel yields of ∼52 000 kg ha^−1^ yr^−1^ compared with ∼150 kg ha^−1^ yr^−1^ for *Zea mays* (maize; corn), ∼860 kg ha^−1^ yr^−1^ for *Brassica napus* (rapeseed; canola) and ∼5000 kg ha^−1^ yr^−1^ for *Elaeis guineensis* (oil palm), although these figures are extrapolations from laboratory experiments and may not scale as anticipated [82].

Oleaginous microalgae such as *Chlorella vulgaris, Scesedesmus spp.* and *Nannochloropsis* have attracted considerable interest as a source of TAG-derived biodiesel [83]. Under nitrogen or phosphate limitation, normal cell division is repressed resulting in excess electrons in the photosynthetic electron transport chain which increases photo-oxidative stress that may damage the photosynthetic apparatus or other cellular components. In response, oleaginous algae divert that excess energy towards fatty acid and TAG biosynthesis, consuming approximately twice the NADPH derived from the electron transport chain than is required to synthesise a comparable mass of carbohydrate or protein [84]. TAGs accumulate in cytoplasmic liposomes or chloroplastic plastioglobuli and can represent 20–50% of the dry cell mass [84]. Levels of TAG accumulation is species- or strain-specific and, despite many screening initiatives, there is little consensus regarding those most suited to TAG-derived biodiesel production [85,86]. That problem is compounded by the fact that microalgal lipid profiles are sensitive to environmental factors and the age of the culture [87] and this variability imposes severe quality control issues and costs for the biodiesel producer. Moreover, despite decades of research into cultivation, processing technologies and life-cycle assessments of microalgal culture [88,89], including the possibilities offered by polyculture to limit population crashes, integrated biorefineries in which the microalgae are used to fix waste CO_2_, purify waste water and produce both high-value chemicals and biomass [90], the translation of laboratory findings to large-scale culture, the upfront capital expenses of suitable land and culture installations at scales that are compatible with fuel production and the operational and downstream processing costs of algal biofuels impose extremely high barriers to investment relative to 1G or 2G biomass. Addressing and overcoming these techno-economic barriers remains an area of intense research [91,92].

### Microalgal hydrocarbons

The green alga, *Botryococcus braunii*, has attracted considerable interest as a possible source of advanced biofuel due to its capacity to both synthesise and secrete 5–80% of its dry mass as C_20_–C_40_ hydrocarbons [93–95] that are readily converted to transport fuels by catalytic cracking [96,97]. *B. braunii* are grouped into four phenotypic races — A [98,99], B [100,101], L [102–104] and S [105] — depending on the hydrocarbons produced and the metabolic pathways employed. In the B race, intracellular liposomes contain ∼7% of total hydrocarbons with the rest located in the algal cell walls and extracellular matrix of the colony [95], a location that favours non-destructive extraction of the hydrocarbons [106]. Despite these considerable advantages, the production of biofuels from *B. braunii* is hindered by the same economic considerations of large-scale algal culture; the costs of the installation, culture and processing still vastly exceed that of the product.

A recent and exciting development in algal biology was the discovery of a light-activated enzyme from the microalga *Chlorella variabilis*, named fatty acid photodecarboxylase (FAP), which catalyses the decarboxylation of free fatty acids to n-alkanes or -alkenes in response to blue light [107]. Engineered into *E. coli* and in conjunction with a thioesterase FAP expression resulted in the production of C_11_–C_17_ hydrocarbons when illuminated by low-irradiance blue light [108].

## Engineering microbial metabolisms for advanced biofuels

While short-chain alcohols, biodiesels and HVOs from 1G and 2G feedstocks are the current solution to fossil fuel mitigation in the transport sector, these are additives that, at high concentrations, compromise fuel quality [109,110]. Direct replacement of fossil-derived base-fuel with straight and cyclic hydrocarbons of varying C-chain lengths and degrees of saturation is therefore desirable [11]. Although the metabolic capacity for hydrocarbon biosynthesis is widely distributed, except for the few oleaginous microbes described above such molecules are produced only in minute quantities (<0.1% of dry mass) and serve primarily physiological rather than energy storage functions, including the regulation of membrane fluidity and permeability, cell–cell signalling, and as a defence against desiccation or environmental toxins [111]. Consequently, the production of direct fuel replacements must be engineered in microbes and would not have been possible without the discovery, characterisation and engineering of enzymes from across the phylogeny [112] such as aldehyde deformylating oxygenases (ADO) from cyanobacteria [113], the ECERIFERUMs (CER) from higher plants [114], insect cytochrome P450's (CYP) [115], the fatty acid decarboxylases P450OleT_JE_ from *Jeotgalicoccus* [116] and UndA from *Pseudomonas* [117] and the FAP [107] that perform the final conversions of endogenous metabolic products to possible fuel molecules. In these investigations, molecules derived from the fatty-acid, terpenoid and polyketide anabolic pathways are typically targeted as substrates for engineering advanced microbial biofuels (Figure 3). These developments owe much to the increased use of Synthetic Biology [118,119] for the design, engineering and iterative development of process-tailored microbes expressing new, proof-of-principle metabolic pathways for the synthesis of potential fuel molecules.

### Engineering fatty-acid advanced derived biofuels

While the enzymes involved are different, fatty acid (FA) synthesis is mechanistically conserved between the prokaryotes and eukaryotes. During FA synthesis, the intermediate metabolites are covalently bound to acyl carrier protein (ACP) by thioester linkages between the carboxyl group of the intermediates and the Ser_36_ of the ACP. FA synthesis occurs in two stages; initiation and cyclic elongation of the C-chain. During initiation of straight-chain FAs, malonyl Co-A is converted to malonyl-ACP which is condensed with acetyl-CoA to produce β-keto-butyryl-ACP. This compound is then reduced to generate D3-hydroxybutyryl-ACP, dehydrated to 2-enoyl-ACP and then reduced to butyryl-ACP. A cycle of condensation with malonyl-ACP and subsequent reduction-dehydration-reduction in the intermediate metabolites builds the C-chain. When the elongating fatty acyl-ACP has achieved a specific length, it is cleaved from the acyl-ACP by thioesterases (TES) to produce free FA (FFA). Overexpression of different, heterologous TES enable FFA tailoring to specific C-chain lengths [11]. Cellular FFA pools are finely balanced between synthesis and degradation (β-oxidation), which initial step is FFA conjugation with acyl-Co by acyl-CoA synthase (FadD) to produce fatty acyl-CoAs and thence acetyl-CoA by degradative thiolases producing metabolic energy. Interestingly, FFA degradation may be reversed (a process termed ‘reverse β-oxidation’) in which the thiolases function as synthetic enzymes, generating long-chain fatty acids directly from acetyl-CoA rather than first requiring malonyl-CoA, thereby increasing the C-efficiency of the overall process [120].

Early metabolic transformations focussed on engineering organisms as catalysts for existing biofuels, for example the production of biodiesel-like compounds in *E. coli* through conversion of free FAs to FAME by heterologous expression of a *Mycobacterium marinarum* fatty acid O-methyl transferase, using endogenous S-adenosylmethionione as the methyl donor [121]. FA metabolism has also been engineered to generate drop-in fuel molecules that are identical with petroleum distillates. FFA can be reduced to fatty aldehydes by the luxCED complex [11] or by CAR [58] and these are then reduced by ADO [113] to alkanes and alkenes of varying C-chain lengths. Either system has its merits, with the luxCED system allowing more refined tailoring of C-chain length and the CAR having a broader range of C-chain selectivity [122]. Alternatively, fatty acyl-ACPs may also be reduced to fatty-aldehydes by an NAD(P)H-dependent fatty acyl-ACP reductase (AAR) [59] and thence to alkanes. Fatty aldehydes and acyl-CoA's may be converted to fatty alcohols, respectively, by aldehyde reductases (AHR) and acyl-CoA reductases (ACR) [123,124]. Finally, as noted previously, a number of other enzymes (e.g. CER, CYP, OleT_JE_ UndA and FAP) have been identified that catalyse the products of lipid biosynthesis to suitable, advanced biofuels. Some bacteria, notably *Bacillus subtilis*, may generate branched chain FAs, in which the initial acetyl Co-A is replaced by a different primer — e.g. isovaleryl-CoA, isobutyryl-CoA, or 2-methylbutyryl-CoA — each derived from the metabolic pathways for valine, leucine and isoleucine. Blending of branched and straight chain molecules allows tailoring and optimisation of the fuels.

While these proofs-of-principle are encouraging, given the levels of global fuel consumption, it is both necessary and possible to increase the titres of biofuels or precursors produced in this manner [125]. For example, TES overexpression has been shown to increase FFA titres [126], possibly by enhancing the ‘pull’ of the metabolic sink, and deletion of FadD, the first enzyme in FA catabolism increases further cellular FFA concentration [127].

### Terpenoid-derived biofuels

Isoprenoids and terpenoids are generated via two alternative metabolisms; the mevalonate and the MEP pathways [128] (Figure 3). In the former, three molecules of acetyl-CoA are condensed via acetoacetyl-CoA to 3-hydroxy-3-methylglutaryl-CoA (HMG-CoA) to mevalonic acid which is the precursor to isopentyl diphosphate (IPP) and dimethylallyl pyrophosphate (DMAPP). IPP and DMAPP are the basic 5-carbon isoprene building blocks for further terpenoid synthesis and undergo a head-to-tail condensation to generate the monoterpene (C_10_) geranyl diphosphate (GPP). Additional condensations with C_5_ isoprenes yield farnesyl pyrophospahate (FPP), the immediate precursor of C_15_ sesquiterpenes and a further isoprene addition forms geranylgeranyldiphosphate (GGPP), the precursor of C_20_ diterpenes. FPP and GPP may also form homodimers by head-to-tail condensation, yielding C_30_ triterpenes and C_40_ tetraterpenes. In the MEP pathway, IPP and DMAPP are formed from the glycolytic cycle.

Terpenoid hydrocarbons are suitable candidates for engineering advanced biofuels due to the branches and rings found in their hydrocarbon chains. The production of C_15_ bisabolane has been performed in *S. cerevisiae* and *E. coli* [129]; that of limonene and oxidosqualene in *Y. lipolytica* [130]; farnesane [131,132] a replacement jet-fuel in *E. coli*; and the tricyclic sesquiterpenes epi-isozizaene and pentalenene in *E. coli*, and α-isocomene in *S. cerevisiae* [133]. While the number of possible terpenoids is vast and represents a daunting bottleneck for further development, recent developments in AI and machine-guided genomic mining hold promise for more rapid identification and testing of possible biosynthetic pathways [134], thereby accelerating and streamlining the biosynthesis of novel biofuels.

### Biofuels from the polyketide synthetic pathway

Polyketides are a structurally diverse group of metabolic products that are synthesised by multienzyme complexes, termed polyketide synthases, in a modular, iterative fashion involving three steps; initiation, elongation and functionalisation [135]. Polyketide synthesis is initiated by condensation of a starter unit (usually acetyl-CoA but can be other metabolic acids/CoA conjugates) with an extender unit, malonyl-CoA or methylmalonyl-CoA. The resulting diketide can then be extended stepwise to form intermediate polyketide chains. The extender units are arranged in a linear series of polyketide synthase modules (PKSs) scaffolded by giant polypeptide chains and have thus been likened to an assembly line. Each PKS in each module consists of an acyltransferase and a ketosynthase. The manner in which the PKS modules are aligned specifies the unique biochemistries of the products generated. The modular nature of polyketide assembly is therefore particularly attractive for re-engineering to generate new compounds [136], of which tailored, drop-in biofuels are a target [137,138].

## Challenges to materiality

Despite the fact that microbes already produce the bulk of (1G) biofuel used worldwide and the increasing body of research into advanced microbial biofuels, the translation from laboratory demonstration to industrial production and commercial distribution is a substantial, multidisciplinary challenge.

While *S. cerevisiae* remains a chassis of choice given its general use within the biofuels industry, the potential of alternative microbial hosts that can metabolise a broader range of substrates are crucial areas for investigation. Further characterisation leading to the eventual ‘domestication’ of these (currently) unconventional microbial chassis [139–141] must therefore be performed in parallel with the development of the molecular tools for engineering robust and predictable metabolic pathways in these candidate hosts [142]. Moreover, as it is improbable that a single, engineered microbe can effectively and simultaneously catabolise complex substrates and produce desired products in large quantities, the possibilities of engineering synthetic microcosms comprising biocatalysts with complementary functions have considerable potential [143,144].

As noted for single-cell oils [68] or microalgal biofuels [81,91], the production and processing costs, not the inherent capabilities of the microorganisms, may be the main impediments to commercialisation [145]. For example, the scale, configuration and operation of the production bioreactor are critical as they dictate the upfront investment, operational costs and the bio-physical environment in which the biocatalyst must thrive, including pH, temperature, [O_2_], type of biomass and possible toxicity of biomass derivatives, waste products and/or of the biofuel itself, and, for non-axenic substrates, competition with the endemic microbiome. Upstream, the selection, harvesting, collection logistics and pre-processing of selected biomass determines the size of the production facility which, in turn will affect the number and geography of the assets and the subsequent distribution networks and the increasingly important impacts of overall carbon-emissions [2,3], changes in land-use or agriculture systems [75], and consequences for global biodiversity [146]. Consequently, new microbial routes to biofuels should routinely be assessed by rigorous stage-appropriate life-cycle and techno-economic assessments (LCA and TCA, respectively) in which the benefits derived from each innovation and the potential costs or diverse impacts of its production and use are clearly stated and quantified [147–149].

## Conclusion

Decarbonisation of the transport sector is essential to enable the transition to net zero C-emissions by 2050 but achieving that aim against a backdrop of increasing energy demand, fragmenting global energy systems and the unpredictable adoption of different technologies is challenging. Microbes display an array of metabolisms from which innovative biofuels may be derived using biomass or other renewable substrates. The engineering of microbial metabolisms by synthetic biology has generated vast new opportunities for the production of advanced biofuels that can replace petroleum distillates. However, translation from laboratory to industry remains a challenge, including the requirement for versatile, readily engineered and industrially compatible microbial biocatalysts with the necessary molecular tools and regulatory sequences, further optimisation of engineered pathways to increase productivity and ongoing techno-economic and life-cycle assessments of these opportunities.

## Perspectives

- Microbes already produce the bulk of commercial biofuel but new, advanced biofuels that can directly replace petroleum distillates are urgently required to accelerate decarbonisation of the transport sector.
- Bacteria, yeasts and microalgae possess extraordinarily diverse metabolisms and the design and engineering of microbial metabolisms by synthetic biology offers great potential for the production of sustainable, advanced biofuels from diverse organic substrates.
- The translation from laboratory to commercial production of advanced biofuels requires a multidisciplinaty effort including the development of versatile, readily engineered and industrially compatible microbial biocatalysts with the necessary molecular tools, further innovation and optimisation of engineered pathways and systematic techno-economic and life-cycle assessments of these new opportunities.

## Abbreviations

AAR: acyl-ACP reductase
ABE: Acetone-Butanol-Ethanol
ACP: acyl carrier protein
ACR: acyl-CoA reductases
ADO: aldehyde deformylating oxygenases
AHR: aldehyde reductases
AMP: adenosine monophosphate
CAR: carboxylic acid reductases
DMAPP: dimethylallyl pyrophosphate
FA: fatty acid
FAP: fatty acid photodecarboxylase
IPP: isopentyl diphosphate
RNG: renewable natural gas
TAG: tri-acyl-glycerides
TES: thioesterases
